# Annual Commencement of the Medical Department of the Buffalo University

**Published:** 1871-02

**Authors:** 


### Annual Commencement of the Medical Department of the Buffalo University.—The Exercises, List of Graduates, etc.

The candidates for graduation received their final examination before the Faculty and Curators, at the College Amphitheatre, Monday morning, Feb. 20, which was sustained with the greatest credit to the class. After the examination, the Faculty, Curators, and Class, retired to the Museum Hall, where was spread an ample and substantial repast, which being served, toasts and sentiments closed one of the pleasant features of the occasion.

It was proposed, by Dr. Strong, of Westfield, that an Alumni Association be formed, as productive of the acquaintance and friendship of the numerous Physicians who now claim Buffalo Medical College as their Alma Mater: as forming a bond of union between them.

All present appeared much gratified at the proposition, and Dr. T. D. Strong, of Westfield, was chosen President, and W. W. Miner, of Buffalo, Secretary. Measures will thus be immediately taken to obtain the names and residences of all who have graduated from the college.

At St. James Hall, in the evening, the attendance was large; and on the stage were members of the Council, Faculty and Curators.

The exercises opened with an overture by Wahle's Band, which was followed by an invocation by the Rev. Dr. Ingersoll. After which the degree of Doctor in Medicine was conferred by the Chancellor, Hon. Millard Fillmore, upon each of the following gentlemen composing the

### GRADUATING CLASS.

Thomas Jackson, M. R. C. S., Buffalo; John Claudius Young, Portville, Cattaraugus county; Andrew Washington Smith, Angelica, Allegany county; George Furguson Dennis, Churchville, Monroe county; Selim Decatur Bouton, Corry, Erie county, Pa.; John Hutchins, Cheshire, Ontario county, N. Y.; Charles Rich Pearce, East Pembroke, Genesee county; Dougal McNeil, Wallacetown, Ontario; Fowler Bradnack, Buffalo; Spurzheim Palmer Moore Bennett's Corners, Madison county; Henry Clay Devening, Buffalo; Rollin

Ledru Banta, Buffalo; DeWitt Clinton Crumb, Preston, Onondaga county; Michael Talbot, Buffalo; Joseph Goffin Bailey, Toronto, Ontario; Frederick William Smith, Laura, Miama county, O.; Devillo White Harrington, Buffalo; John E. McTaggart, Bridgetown, Ont.; Edward John Brennan, Buffalo; Albert Henry Briggs, Elma, Erie county, N. Y.; James Fennimore Cooper, Hornellsville, Steuben county; James Henry Trumbull, Hornellsville, Steuben county; Oscar Franklin Decker, West Falls, Erie county, N. Y.; Theophilus Stewart Hartley, A. M. Hankins, Sullivan county, N. Y.; Arthur Hamilton Smith, Rochester, N. Y.; Solomon Jennings, West Milton, Miami county, O.; Oscar Seth Pratt, Byersville, Livingston county; John Stone Perkins, Charlestown, Middlesex county, Mass.; Mortimer Cherberry Bissell, Lyndon, Cattaraugus county; James Polk Rathbun, Weatherfield, Wyoming county; Charles Mills Stewart, Hume, Allegany county; Worthington Warner Miner, A. B., Ware, Hampshire, Mass.; Otis Allen, New Hudson, Allegany county, N. Y.; John Joseph Walsh, Buffalo; Stephen Albert Russell, Fredonia, Chatauqua county; Silas Wright Robinson, Nunda, Livingston county; Orville Clarke Strong, Colden, Erie county.

Following the conferring of degrees came the Address before the Graduating Class by Prof. E. M. Moore, which is of such scope and finish that we propose to furnish it to our readers as early as possible.

At the close of the address the benediction was pronounced by Rev. Dr. Heacock.

On recommendation of the Faculty and Curators the council conferred the honorary degree of Doctor in Medicine on Dr. Edward Smith, of Lewiston, Niagara county, N. Y.; and Dr. George Mann, of Newfane, Niagara county.

The Faculty and Curators directed that the Thesis of W. W. Miner, on Excisions involving the joints of the upper extremity, receive honorable mention and be recommended for publication. Also, that the Thesis of D. W. Harrington, on Uraemia, and that of H. C. Devening, on Phlegmasia Dolens, receive honorable mention.

The following Curators were present at the examination, and on the stage in the evening: Dr. Lewis P. Dayton, Dr. J. B. Samo, Dr, C. C. Wyckoff, of Buffalo; Dr. George P. Eddy, Lewiston, Niagara county, N. Y.; Dr. Matthew S. Moore, Fredonia, Chautauqua county, N. Y.; Dr. Robert J. Menzie, Caledonia, Livingston county, N. Y.; Dr. J. W. Craig, Churchville, Monroe county, N. Y.; Dr. Morris W. Townsend, Bergen, Genesee county, N. Y.; Dr. P. H. Flood, Elmira, Chenango county, N. Y.; Dr. M. E. Potter, Attica Wyoming county, N. Y.; Dr. T. D. Strong, Westfield, Chautauqua county, N. Y.; Dr. W. McCollum, Lockport, Niagara county, N. Y.; Dr. W. B. Gould, Lockport, Niagara county, N. Y.; Dr. G. H. Lapham, Aurora, Erie county, N. Y.; Dr. Harvey Jewett, Canandaigua, Ontario county, N. Y.; Dr. E. E. Fuller, Fredonia, Chautauqua county, N. Y.

The following members of the Council were also present: O. H. Marshall, Esq., President; Hon. E. G. Spaulding; Hon. Orlando Allen; John D. Shepherd, Esq.; George S. Hazard, Esq.; Dr. George E. Hayes.

### *Annual Report of the Surgeon General of the U. S. Army*, 1870.

The present condition of the medical department of the United States Army is one of prosperity and efficiency. We have good reason to believe that its affairs have been under the care of an able and energetic administration. While the sanitary condition of the army does not vary materially from what it was during the year preceding, the amount of scientific information and facts, which the department is attaining and promulgating, is of continually increasing interest and utility. The report is for the fiscal year ending June 30th, 1870, from which it appears that the annual appropriation made by Congress for the Medical and Hospital department of the Army was $247,000, of which there remains an unexpended portion of ten thousand dollars, while the amount of unexpended funds in the hands of the department is over a milion and a half dollars. Immediately succeeding the tabular statement of finances is the sanitary report, portions of which are here given:

"The average number of white men constantly on sick report was 1,419; of these 1,156 were under treatment for disease, and 263 for wounds, accidents and injuries. The total number of deaths was 374; of these 249 died of disease, and 125 of wounds, &c. The comparatively large mortality from wounds is explained by the Indian hostilities which continue to exist.

The proportion of deaths from all causes to cases treated was 1 death to 167 cases; 745 were discharged on certificates of disability. In colored troops the average number constantly on sick reports was 178, of whom 146 were under treatment for disease, and the remainder for wounds, &c. The number of deaths from all causes was 66, of whom 51 died of disease, and 15 from wounds, &c. The number discharged for disability was 104. In addition to the large amount of clerical labor performed, 3,029 photographs were printed; 106 wood cuts were made; 153 pages of the Surgical History; 272 pages of the appended documents to the Medical and Surgical History; 59 histories of photographs, or abstracts of cases to accompany photographs, were printed.

The printing of the medical volume of the first part of the Medical and Surgical History of the War is near completion. This volume embraces the statistical tables representing the sickness, mortality and discharges from service on surgeon's certificate of disability, of white and colored troops during the war, and will be a work of nearly 750 pages quarto.

There has been a very steady and uniform increase in the various collections of the Army Medical Museum. The Indian hostilities, and the accidents of the field and camp and garrison, have afforded the opportunity of collecting some illustrations of the injuries inflicted by weapons—a class of specimens in which the Museum is already surpassingly rich—but the more numerous contributions to the surgical section have been of specimens illustrating pathological processes, or the remote effects of injuries.

As the date of the last annual report, 2 vacancies in the grade of surgeon, and 42 in that of assistant surgeon United States army, existed. During the past year 4 assistant surgeons have resigned, and as the act of Congress, dated March 3d, 1869, still continues in force, no vacancies have been filled—total number of vacancies at the present time, 2 surgeons and 46 assistant surgeons. The number of commissioned medical officers available for duty with troops on the 30th of June, 1870, was 147; on leave of absence, 4; on sick leave, 4. The estimated number of troops in service at that period was 32,429. There were 217 military posts, besides numerous detachments serving in the field and on outpost duty, each requiring a medical officer. The number of commissioned medical officers being inadequate, contract surgeons are employed, as heretofore; but it would be more economical and satisfactory to be able to fill the existing vacancies in the regular medical staff of the army. So long as our extended frontier exists with its isolated military posts and moving detachments of troops, so long will the medical staff be required to be kept up to the standard number allowed by existing laws, and any reduction of that number will be prejudicial to the best interests of the military service."

